# Flexible and Compressible Nanostructure-Assembled Aramid Nanofiber/Silica Composites Aerogel

**DOI:** 10.3390/ma17091938

**Published:** 2024-04-23

**Authors:** Chensi Zhang, Jiangtao Li, Junpeng Jiang, Xiaoxia Hu, Shuo Yang, Kuan Wang, Anran Guo, Haiyan Du

**Affiliations:** 1 School of Materials Science and Engineering, Key Lab of Advanced Ceramics and Machining Technology of Ministry of Education, Tianjin University, Tianjin 300072, China; zcs990513_@tju.edu.cn (C.Z.); jiangtaoli@tju.edu.cn (J.L.); jpjiang@tju.edu.cn (J.J.); kuanwang@tju.edu.cn (K.W.); arguo@tju.edu.cn (A.G.); 2 Analysis and Test Center, Tianjin University, Tianjin 300072, China; xiaoxiahu@tju.edu.cn (X.H.); shuoy@tju.edu.cn (S.Y.)

**Keywords:** aramid nanofiber, impregnation duration with silica sol, mechanical flexibility and robustness, surface modifier, thermal insulation

## Abstract

The Applications of silica aerogel are limited due to its brittleness and low strength. As a result, it is essential to strengthen and toughen it. Organic nanofibers are one of the preferred reinforcement materials. In this work, we designed and fabricated flexible and compressible nanostructure-assembled aramid nanofiber/silica composites aerogel (ANF/SiO_2_ aerogel) to improve the mechanical strength and flexibility of silica aerogel without compromising thermal insulation properties. The aramid nanofiber/silica composite aerogels were prepared by immersing the aramid nanofiber wet gel into the silica sol for a certain period of time followed by freeze drying without solvent replacement. The surface modifier 3-aminopropyltriethoxysilane (APTES) was used as a coupling agent to form chemical linkage between the ANF fiber and silica gel. It was observed that APTES can effectively drive the silica sol to infuse into ANF hydrogel, promoting the assembly of silica gel onto the fiber surface and a uniform distribution in the network of ANF. The compressive resilience, thermal stability, and thermal insulation properties of the composite aerogels were evaluated by inducing the silica aerogel into the ANF network to form a protective layer on the fiber and change the pore structure in the ANF network.

## 1. Introduction

Silica aerogel has a unique continuous three-dimensional nano-network structure, featuring an ultra-high specific surface area and porosity, a very low density, low thermal conductivity, a low refractive index, and other excellent characteristics [1,2,3,4]. Therefore, the potential application of silica aerogel has widened beyond its use in various industrial areas such as weaponry, aerospace, industrial energy saving, building energy saving, petrochemical, and heat protection applications [5]. However, the application of silica aerogel is limited by its poor mechanical properties, low strength, and high brittleness [6]. Based on current research, the use of fiber material in the manufacture of a SiO_2_ aerogel composite is an effective method of overcoming the mechanical limitations of silica aerogel.

Inorganic fibers, such as mineral [7], ceramic [8,9], and glass fibers [10,11], are the main components of fiber/silica aerogels. While the addition of inorganic fibers can improve compressive strength, it often comes at the cost of sacrificing the advantages of low density and low thermal conductivity due to the brittleness of the fibers themselves. Inorganic fibers are not suitable for withstanding bending stresses and have a high density [7,12]. Over the past few decades, hybrid and composite materials based on polymers and inorganic materials have gained a lot of attention, not only for their interesting structural characterization, but also for their promising functional applications. Shaghayegh Hamzehlou et al. have introduced an elegant selection of first-rate reviews and original research articles on the design and performance of multifunctional hybrid materials based on polymers [13]. In the works about hybrid materials based on polymers and inorganics (including nanomaterials), the chemical combination of polymers and inorganic components is a key issue that needs to be addressed when making polymer–inorganic composites [14]. Xuan Wang et al. [15] have developed functionalized SiO_2_/XLPE nanocomposites by chemically grafting auxiliary crosslinkers onto nanosilica surfaces. Trimethylolpropane triacrylate (TMPTA) is successfully grafted on nanosilica surfaces through thiolene click chemical reactions. With the coupling agents of sulfur silane and 3-mercaptopropyl trimethoxy silane (MPTMS), the functionalized SiO_2_ nanoparticles could be dispersively filled into polyethylene matrix even at a high filling content.

In recent years, there has been a growing interest in using silica composites with organic fibers to create flame-retardant and high-temperature anti-insulation materials. The aerogel and organic fiber composite system has been found to have more elasticity and flexibility, lower density, and better thermal insulation compared to inorganic fiber composites. In the selection of organic fibers, it is important to take into account their thermal stability, which should be higher than that of the pure silica aerogel. Tetraethoxysilane (TEOS)-based aerogels exhibit thermal stability within the range of 300 °C to 330 °C [16,17]. Aromatic polyamide fibers are widely recognized as excellent organic fibers due to their low density, low thermal conductivity, and high mechanical strength, making them a popular choice for reinforcement purposes. As stated in the Kevlar technical brochure, aramid fibers can withstand pyrolysis in air at temperatures of up to 430–480 °C, so the selection of temperature-resistance conditions has been met. Furthermore, aramid fibers possess rigid molecular chains and good extensibility, interacting through a robust hydrogen bonding network. This results in excellent mechanical properties, high chemical resistance, and flame retardancy, making them ideal for reinforcing silica aerogels while also providing flexibility [18]. The thermal insulation properties of various materials prepared from aramid nanofibers have also been reported, with the composite film KNA/PCM having a thermal conductivity of 0.036 W m^−1^ k^−1^ at room temperature [19], and an ANF aerogel prepared with the auxiliary molding agent PVA having a thermal conductivity of 25.78 mW m^−1^ k^−1^ at room temperature; it can be heated on a heating plate at 200 °C for 500 s to maintain its elasticity [20]. In addition, ANF aerogel fibers are woven into textiles to illustrate their excellent thermal insulation property under extreme temperature (−196 or +300 °C) and at room temperature [21]. As a result, aramid fibers were chosen as the reinforcement material in this study.

Previously, there have been reports on composite aerogels made of silica nanoparticles reinforced by aramid fibers, pulp, or fabric [22,23,24,25]. Encouraging results have been presented, showing that the composites exhibit low density, low thermal conductivity, high thermal stability, and high compressive strength. As a result, further research is needed to investigate the flexibility and compressive resiliency of aramid fiber-reinforced silica aerogel, as there has been insufficient research on this topic previously. In this work, the flexible aramid nanofiber network (ANF) was employed for assembly with silica aerogel to overcome brittleness and gain enough load-bearing capacity by means of the strong interaction and anchoring between the nanofibers and silica aerogel. The surface modifier 3-aminopropyltriethoxysilane (APTES) was used as a coupling agent to enhance the chemical grafting between the ANF fiber and silica gel. The effect of APTES and SiO_2_ aerogel contents on the structure and mechanical and thermal properties of the ANF/silica composite aerogel were investigated.

## 2. Materials and Methods

### 2.1. Raw Materials

Kevlar fibers were purchased from DuPont Ltd., Wilmington, DE, USA. Dimethylsulfoxide (DMSO) (>99.8%, GC); potassium hydroxide (99.99% metals basis), cetyltrimethylammonium chloride (CTAC) (97%), and 3-aminopropyltriethoxysilane (APTES) (98%) were obtained from Aladdin (Shanghai, China). Anhydrous ethanol (AR), ammonia (25–28 wt%), and acetic acid (99.9%) were purchased from Tianjin Komeo Chemical Reagent Co., Tianjin, China. Methyltrimethoxysilane (MTMS) (98%) was obtained from Shanghai Eon Chemical Technology Co. Shanghai, China. Tert-butanol (GC) was sourced from Shanghai McLean Biochemical Technology Co. Shanghai, China. Additionally, 5 mM HAc and 1 mol/L NH_4_OH prepared by dilution with deionized water were used as the acid and base catalysts, respectively.

### 2.2. Preparation of ANF/Silica Aerogels

**Figure 1:**
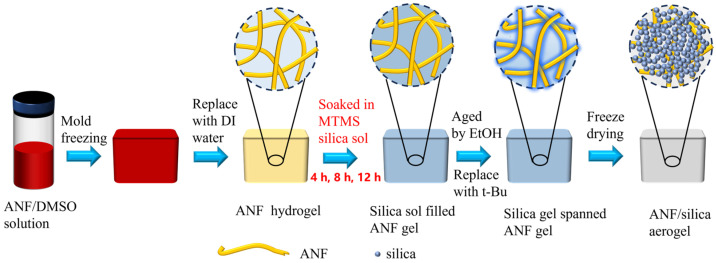
Schematic diagram of the preparation process of composite aerogel.

A schematic flow chart of the composite aerogel preparation process is shown in Figure 1.

#### 2.2.1. Preparation of Aramid Nanofiber Solutions

The literature reports that aramid nanofibers can be dispersed in dimethyl sulfoxide (DMSO) either by adding a plasmon donor or through physical fibrillation [26]. For this experiment, 0.25 g of potassium hydroxide (KOH) and 1.5 g of Kevlar fiber were added to 125 mL of DMSO. The mixture was then stirred at 4000 rpm for 5 min, resulting in a macroscopically homogeneous yellowish aramid fiber premix. A blend of the premix and 5 mL 10 wt% KOH solution was achieved by mechanically stirring for 4 h in ambient temperature, obtaining a homogeneous and transparent dark red solution of ANF/DMSO. Subsequently, 5 g ANF/DMSO solution and 5 g 0.3 wt% APTES/DMSO solution were blended and stirred for 20 min to obtain a uniform APTES/ANF/DMSO solution.

#### 2.2.2. Preparation of ANF Hydrogel

To facilitate the preparation of an ANF hydrogel, the ANF/DMSO frozen blocks were prepared by pouring the APTES/ANF/DMSO solution into some molds and freezing them at −60 °C for 20 min. The ANF hydrogels were obtained by immersing the frozen blocks into deionized water for 36 h and changing the water three times during this period.

#### 2.2.3. Preparation of Silica Sol

MTMS was slowly added to a mixture of acetic acid (HAc) aqueous solution (5 mM) and CTAB at room temperature. The molar ratio of MTMS:HAc:CTAC was fixed at 1:1.52:9.54 × 10^−3^. After the mixture was stirred for 30 min, NH_4_OH solution was added and stirred for another 10 min to obtain a hydrolyzed MTMS solution. The molar ratio of MTMS:NH_4_OH was 1:0.004.

#### 2.2.4. Preparation of ANF/Silica Aerogel

The ANF hydrogels were impregnated with the silica sol for 4, 8, and 12 h, respectively, and then placed in an oven at 60 °C until they gelled. The composite gels were soaked in ethanol and aged at 60 °C for 24 h. Subsequently, the ethanol was completely replaced by tert-butanol as a freezing agent within 48 h (the tert-butanol was replaced every 12 h). The composite wet gels impregnated by tert-butanol were quickly frozen in a refrigerator at −60 °C and then freeze-dried under a vacuum of 45 kPa for 36 h to obtain the composite aerogels. Different composite aerogels were prepared by setting the silica sol impregnation time to 4, 8, and 12 h, and these conditions were named Com-4, Com-8, and Com-12, respectively.

### 2.3. Characterization

The bulk density of the ANF/silica composite aerogel was calculated according to the following formula:

ρ = m/V.(1)

The microstructure of the aerogel was investigated using field emission scanning electron microscopy (S-4800, Hitachi, Tokyo, Japan). The composite aerogel’s specific surface area and pore size distribution were determined by using nitrogen suction desorption (Tristar II 3020 M, Micromeritics Instrument Corporation, Norcross, GA, USA). Fourier Transform Infrared Absorption Spectroscopy (Nicolet, Seymour Fisher Technology (China) Co., Shanghai, China) was used to determine the infrared spectra in the wavelength range of 500–4000 cm^−1^ and to characterize the chemical structure of the nano-aramid fibers before and after modification. The mechanical properties of the composite aerogels were determined through a uniaxial compression–decompression test (CMT4304, Meters Industrial Systems (China) Co., Shanghai, China) using a universal testing machine with a set compression rate of 2 mm/min. The samples’ thermal stability was determined by measuring their weight loss from room temperature to 1000 °C under air at a heating rate of 10 °C/min using DTA-DSC (STA 449F3, NETZSCH Instrument Manufacturing Corporation, Selb, Germany). Room temperature thermal conductivity was measured using a thermal constant analyzer TPS2500S (Hot Disk^®^, Gothenburg, Sweden). Thermal imaging was recorded with a thermal imaging camera (LT7-P, Zhejiang Dali Technology Co., Hangzhou, China), and the aerogel surface temperature data were analyzed using Fluke Connect analysis software (https://www.fluke.com/).

## 3. Results and Discussion

### 3.1. The Microstructure of Aramid Nanofiber/SiO_2_ Composite Aerogels

#### 3.1.1. Effect of the Incorporation of Modifier APTES

The hydrophilicity of aramid nanofibers obtained after deprotonation is poor since there are no polar groups on the surface [27]. Surface modification is required to improve the bonding between the fiber and the silica sol. Thus, 3-aminopropyltriethoxy is often regarded as a good cross-linking agent for synthesizing silica aerogels doped with organics [28,29]. In this work, 3-aminopropyltriethoxysilane (APTES) was used as a modifier to promote the absorption of silicon hydride bonds with aramid fiber as well as the uniform distribution of silica sol in the fiber network.

**Figure 2:**
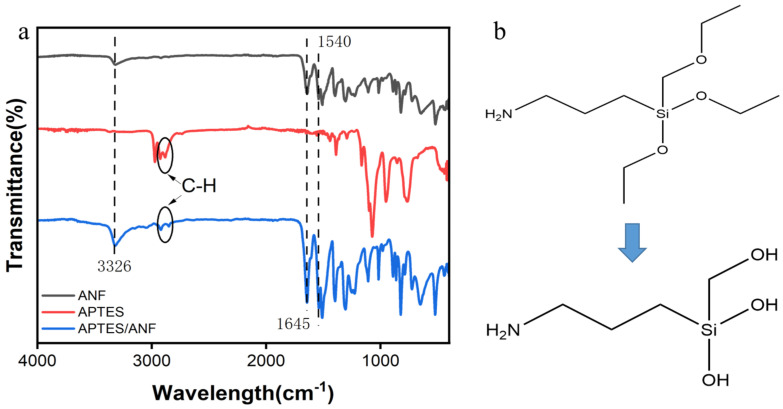
(**a**) FT–IR spectra of ANF aerogel, APTES, and APTES/ANF aerogel and (**b**) molecular formula of APTES before and after hydrolysis.

FT–IR analysis was conducted on the ANF before and after APTES treatment (Figure 2a). It is shown that both the ANF and APTES/ANF spectra displayed the typical characteristic peaks of amide groups of aramid nanofibers in Figure 2a. The absorption peaks at 3326 cm^−1^, 1645 cm^−1^ and 1540 cm^−1^ are attributed to the -N-H- stretching vibration, -C=O stretching vibration and -N-H- bending, respectively [25,30]. However, compared with the ANF spectral line, the APTES/ANF spectral line obviously has two more peaks, 2920 cm^−1^ and 2852 cm^−1^, which are exactly the expansion and contraction vibration peaks of -C-H- in the methylene group in the APTES spectral line [29]. This indicates that APTES successfully modified the aramid nanofibers. The structural formulas of pristine and hydrolyzed APTES are illustrated in Figure 2b. It is shown that the silica hydroxyl group was formed by dehydrogenation of the ethoxylate bond of APTES, which is the key to improving the hydrophilicity of the fibers.

**Figure 3:**
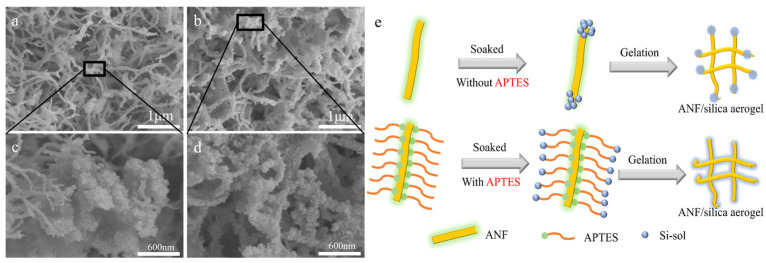
Scanning electron micrographs of ANF/silica composite aerogels (**a**,**c**) without APTES; (**b**,**d**) with APTES, and (**e**) schematic representation of the silica sol impregnation and gelation process within the ANF aerogel framework without APTES and with APTES.

Figure 3 shows the microstructure of the ANF/silica composite aerogel before and after modification. The silica aerogel particles were filled into the ANF gel network, as demonstrated in Figure 3a,b, regardless of whether the modifier APTES was added or not. However, due to the introduction of APTES, the silica aerogel particles were more uniformly distributed on the fiber surface and between the pores, as shown in Figure 3b,d. In contrast, the unmodified samples showed a concentration of silica aerogel particles at the ends and laps of the fibers (shown in Figure 3a,c). Figure 3e illustrates a schematic diagram of the modification principle of APTES on the ANF surface and its influence on the formation of composite aerogel structures. It is indicated that when APTES is mixed with ANF in solution, the aminopropyl end of APTES binds to the surface of the fibers, while silica hydroxyls remain suspended in the aqueous solution. When the APTES/ANF gel network is immersed in the silica sol for solvent replacement, the silica hydroxyl groups in the sol preferentially interact with the suspended silica hydroxyl groups formed by the hydrolyzed APTES coated on the ANF. Subsequently, the silica sol uniformly filled in the pores of the ANF network. In contrast, the hydrophobic surface of the ANF exhibits a low affinity for the silica sol groups. As a result, the silica sol tends to accumulate at the endpoints of the fibers, leading to an inhomogeneous structure.

#### 3.1.2. Effect of Impregnation Duration of ANF Hydrogel with Silica Sol

The ratio of silica to ANF in the composite aerogel structure was adjusted by immersing the ANF hydrogel in the silica sol for different times. The impregnation durations were chosen to be 4 h, 8 h and 12 h, respectively. The mass fractions of silica in the composite aerogel were 90%, 92%, and 95%, and were calculated by using the following formula:

s = 1 − m_0_/m. (2)

where s is the mass fractions of silica aerogel, m_0_ represents the weight of nano-aramid fibers, and m is the weight of the composite aerogel. Furthermore, the APTES/ANF/silica ratios of the resulting materials were 1:4:45, 1:4:57.5, and 1:4:95.

**Figure 4:**
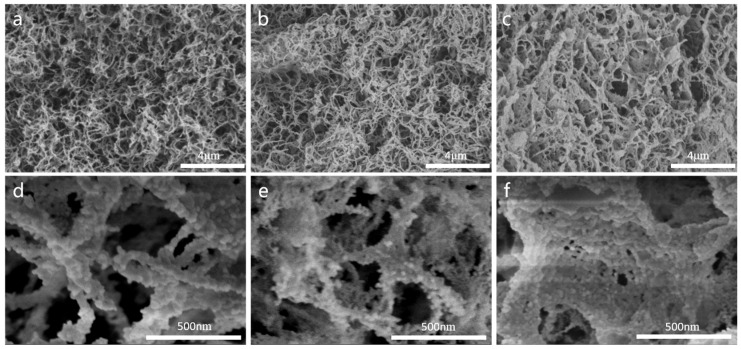
SEM images of composite aerogels (**a**,**d**) Com-4; (**b**,**e**) Com-8, and (**c**,**f**) Com-12.

By comparing the SEM image shown in Figure 4a–c, the composite aerogels can all be observed as uniform fiber network structures, indicating that we have successfully constructed aramid fiber templates. As the impregnation duration of silica sol was extended from 4 h to 12 h, the agglomerated silica aerogel was observed more and more clearly, which was also reflected in the fact that the calculated aerogel content in the samples gradually increased. This indicates that the ANF/silica aerogel became more internally compact as the impregnation time increased; thus, it can be inferred that a further extension of the impregnation time does not lead to major changes in the structure of the composite aerogel. It is evident that when the impregnation time is shorter (4 h), the silica aerogel particles mainly adhere to the surface of the ANF fibers, and the single fiber compounded with particles gradually increases from 41.8 nm to 62.7 nm (Figure 4d–f). As the impregnation time prolongs, silica aerogel particles gradually fill into the network pores, and the large pores gradually decrease.

**Figure 5:**
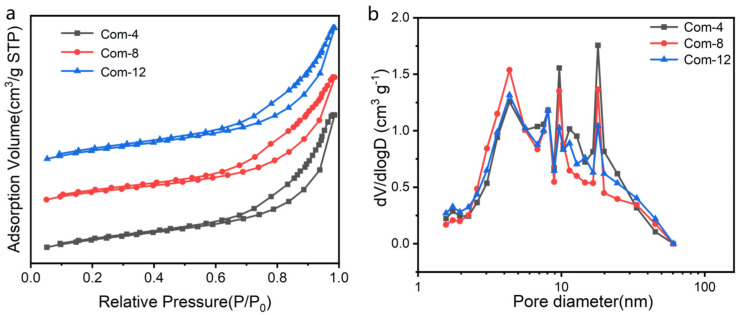
(**a**) Nitrogen adsorption–desorption curves and (**b**) BJH pore size distribution of composite aerogels Com-4, Com-8, and Com-12.

Figure 5a shows that the adsorption/desorption isotherms of the composite aerogels Com-4, Com-8, Com-12 are the typical type Ⅳ form according to IUPAC classification. Meanwhile, the curves show the muddy pore with the H3 hysteresis loop, which is characteristic of mesoporous materials [31]. In type H3 loops, the ascending and descending boundary curves are sloping, and usually the desorption branch also includes a steep region at which the remaining condensate comes suddenly out of the pores as a consequence of the so-called tensile strength effect. Although the curves in the pore size distribution graph given in Figure 5b are not single peaks, the range of their appearing pores falls in the mesoporous interval of 2–50 nm, which also confirms that the obtained composite aerogel materials are indeed mesoporous. The pore size distribution graph indicates that the silica aerogel’s pore size has a higher probability of occurring in the small size range as the impregnation time increases. Comparing Com-4, Com-8, and Com-12, the peaks of Com-4 at 9 and 18 nm are sharper and more intense, so the average pore size of 5.588 nm derived from the BJH calculation method is also the largest, as shown in Table 1. Com-8, on the other hand, appears as fewer peaks and has the highest peak intensity at 4 nm, so its average pore size is numerically slightly smaller than that of Com-12. Table 1 also presents additional physical property data of the composite aerogels. The specific surface area increases as the silica sol impregnation time increases during the preparation process. This is mainly due to the increase in silica aerogel. The network pores with large pore size are filled by silica aerogel with nano pore size, which increases the overall amount of nano pores. As a result, the specific surface area increases with the same porosity.

### 3.2. Mechanical Robustness and Flexibility

**Figure 6:**
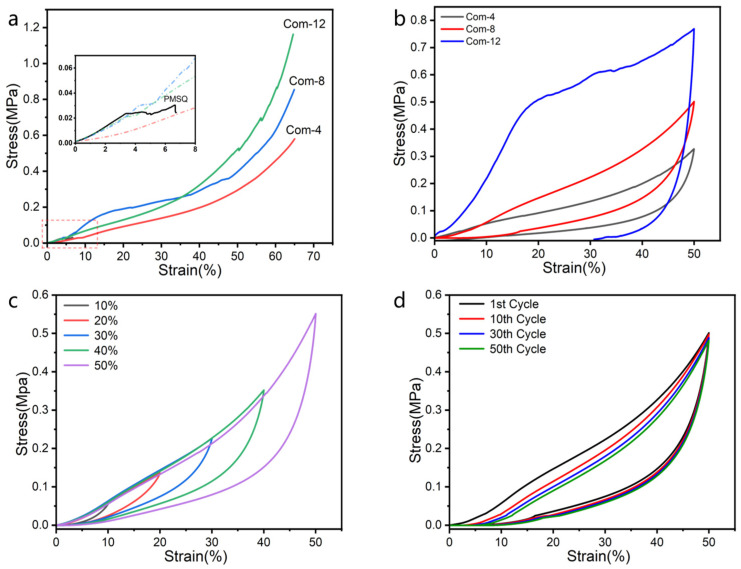
Stress–strain curves of composite aerogels: (**a**) stress–strain during loading of PMSQ and composite aerogels, inset shows the enlarged image of stress–strain during loading of PMSQ, (**b**) stress–strain during loading and unloading of the composite aerogels, (**c**) compression-resilience property of Com-8 at different compression strains, and (**d**) compression-resilience property of Com-8 at 50% strain for 50 cycles.

The stress–strain curves of pure silica aerogel and the composite aerogels were obtained at a loading rate of 0.5 mm min^−1^ by using a universal tester. As shown in Figure 6a, the Com-12 with higher silica content exhibited a higher maximum stress at 60% strain (1107 ± 116.3 kPa), which is about 30 times higher than that of the pure SiO_2_ aerogel (PMSQ) shown in the local enlarged drawing in Figure 6a. The compression rebound curves of the composite aerogel are shown in Figure 6b. The figure shows that when pressure is released, the Com-4 and Com 8 recover to almost their original size, while the Com 12 retains nearly 30% of deformations permanently. The compressive stress at 50% strain increases as the mass of silica increase in the composite aerogel. The loading curves for Com-12 can be divided into three regions: elastic stage, yielding stage, and densification stage (elastic-plastic stage). Starting from the linear stage (strain values between 0–20%), the compression curve slope remains almost constant. The main load-bearing in this stage is due to the open pores of the ANF and the elastic bending of the pore walls. During the yield stage (18–25% strain), stress increases nonlinearly. The main load-bearing comes from the nanopore structure of the silica aerogel, while the fibers act as a supporting skeleton, dispersing and transmitting the external force to the entire sample, which prevents the collapse of the composite aerogel’s overall structure [25]. During the densification stage (30–50% strain), the slope of the stress–strain curve is significantly lower than that of the elastic stage. This is due to the densification of the porous structure of the silica aerogel and the gradual compaction of the aramid nanofibers, which work together to resist the compressive stress [32]; most of deformation at this stage cannot be recovered. Com-8 exhibits almost 100% resilience at 50% strain, with a better elastic modulus than that of Com-4. Thus, Com-8 was chosen for the verification of flexibility, as shown in Figure 6c,d. After 50 cyclic fatigue tests at a set strain of 50%, Com-8 still maintained 90% resilience with only a 1.6% mass loss and without significant damage to the overall structure. The above results indicate that the ANF/SiO_2_ composite aerogel exhibits excellent compression resistance and good resilience.

### 3.3. Thermal Stability Analysis

In order to evaluate the thermal stability of the composite aerogel materials, differential thermogravimetric analysis (TG-DSC) was performed on the ANF aerogel, silica aerogel, and Com-8 composite aerogel, respectively, from room temperature to 1000 °C under air atmosphere. A small amount of poly (vinyl alcohol) (PVA) was added to ensure that the ANF aerogels did not shrink drastically during lyophilization.

**Figure 7:**
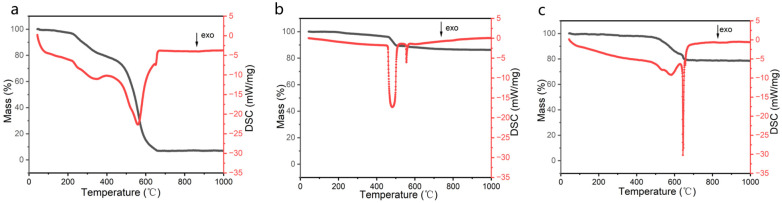
TG-DSC curves of (**a**) ANF aerogel, (**b**) silica aerogel, and (**c**) Com-8 composite aerogel.

The thermal weight loss of the ANF aerogels between 225 and 350 °C, as shown in Figure 7a, was attributed to the decomposition of the hydroxyl groups in the PVA macromolecules, which accounted for approximately 14.5% [20]. The second mass loss occurred at approximately 400 °C, when the ANF began to decompose gradually. The fibers then decomposed violently at 525 °C, releasing a large amount of heat. At 650 °C, 71.2% of the mass was lost, leaving only 7.9 wt% residual carbon. The TG–DSC curve in Figure 7c shows that the sample undergoes a thermal weight loss of ~12.9% between 480 and 620 °C, and it exhibits two exothermic peaks at 580 °C and 644 °C. The oxidation of the hydrophobic Si-CH_3_ group on the surface of the silica particles [33] and the decomposition of the nano-aramid fibers are embodied in this process, which can be matched with the two exothermic peaks mentioned above [34]. The exothermic peak observed at 644 °C, which corresponded to a 3.7% weight loss, could be attributed to the secondary decomposition of methyl, resulting in the removal of silica pore defects [25]. The composite aerogel exhibited improved thermal stability due to the synergistic interaction of the nano-aramid fibers with the silica aerogel. Regarding the Com-8 composite aerogel, the structure was designed with fibers as the backbone and silica being loaded onto it. The outer shell of the nano-aramid fibers protects the methyl group on the surface of the silica from exposure to the outside air in a large area. In turn, the silica protects the nano-aramid fibers from undergoing ablation too quickly [22,25,32].

### 3.4. Thermal Insulation Performance Analysis

**Figure 8:**
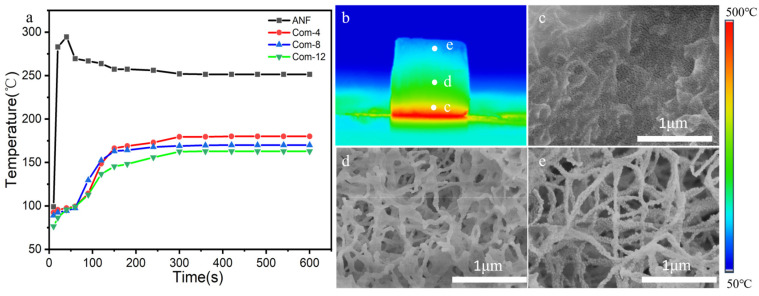
(**a**) Thermal images of ANF aerogel and composite aerogel tip temperature versus time plots; (**b**) composite aerogel Com-8 heated at 500 °C on a heating table for 1800 s and (**c**–**e**) SEM of the aerogel structure from the hot end to the cold end after 1800 s of one-sided heating.

The room temperature thermal conductivities of the ANF aerogels and the composite aerogels Com-4, Com-8, Com-12 are shown in Table 1. The thermal conductivity of the composite aerogels is higher than that of the ANF aerogel due to the effect of solid-phase heat transfer resulting from their higher density. However, only the composite aerogel samples were compared, and after the silica aerogel content increased, the nanopore content increased, which restricted the thermal movement of air molecules and limited the convective heat transfer with little change in density, so that the thermal conductivity of the composite aerogel instead decreased with the increasing impregnation duration [35]. To evaluate the thermal insulation performance of composite aerogels at higher temperatures (500 °C), the ANF aerogels and the composite aerogels of Com-4, Com-8, and Com-12, each with a thickness of 10 mm, were placed on a heating table at 500 °C. The temperature curves of the top surface, 9 mm from the bottom of the hot end, were recorded as a function of time. Figure 8a illustrates that the ANF experienced a sudden increase in temperature and emitted white smoke, indicating that the bottom surface in direct contact with the bottom plate was burnt. The top temperatures of all the composite aerogels increased over time and reached a thermal equilibrium state after 5 min. Comparing the values of the equilibrium temperatures obtained after 5 min of heating for the ANF aerogels and the composites Com-4, Com-8, Com-12, it is clear that silica aerogel plays a significant role in thermal insulation in composite aerogels. The equilibrium temperatures of Com-4, Com-8, and Com-12 also decreased gradually with the accumulation of silica particles, which also matched with the decrease in the thermal conductivity values of the composite aerogels tested before.

The microstructure of Com-8 at different positions from the hot side to the cold side is shown in Figure 8. The positions are labelled c, d, and e in Figure 8b, which shows it after being heated for 1800 s on one side. Figure 8c illustrates that the organic fibers located at the hot end of the aerogel decomposed, while the silica aerogel structure was destroyed. Fibers near the middle of the aerogel also showed damage but had not yet completely decomposed, as shown in Figure 8d. The structure of the top was relatively intact (Figure 8e). Furthermore, the composite aerogels experienced a minor mass loss of approximately 13.7% when they were exposed to a unilateral heating table at 500 °C for 2 h. In conclusion, the complex aerogel materials we prepared demonstrate excellent insulation properties and high thermal stability under 500 °C.

## 4. Conclusions

In this work, an ANF/silica composite aerogel was fabricated via a straight forward in situ synthesis strategy in which an ANF network was selected as a template for the growth of a silica aerogel network. The aramid nanofiber/silica composite aerogels were prepared by immersing the aramid nanofiber wet gel into the silica sol for a certain period of time and this was followed by freeze drying without solvent replacement. The surface modifier 3-aminopropyltriethoxysilane (APTES) was used as a coupling agent to form chemical linkage between the ANF fiber and silica gel. The result shows that APTES can effectively drive the silica sol infusing into ANF hydrogel and promote the assembly of silica gel onto the fiber surface as well as induce uniform distribution in the network of the ANF. The compressive resilience, thermal stability, and thermal insulation properties of the composite aerogels were evaluated by inducing the silica aerogel into the ANF network to form a protective layer on the fiber, changing the pore structure in the ANF network.

## Figures and Tables

**Table 1:** Basic parameters of ANF/silica aerogel samples.

Samples	Silica Mass (%)	Specific Surface Area (m^2^/g)	Average Pore Size (nm)	Thermal Conductivity (mW m^−1^ K^−1^)	Compressive Stress at 60% Strain (MPa)
Com-0	0	253.58	25.31	30 ± 3	0.12
Com-4	90%	391.97	5.588	41 ± 2	0.52
Com-8	92%	397.33	5.169	34 ± 3	0.64
Com-12	95%	418.32	5.202	30 ± 3	1.10

## Data Availability

Data are contained within the article.
